# Origin of Some Common Plants

**Published:** 1881-11

**Authors:** 


### ORIGIN OF SOME COMMON PLANTS.

Cabbage grew wild in Siberia; celery originated in Germany: the potato is a native of Peru; the onion originated in Egypt; tobacco is a native of South America; millet was first discovered in India; the nettle is a native of Europe; the citron is a native of Asia; oats originated in North Africa; rye came originally from Siberia; parsley was first discovered in Sardinia; the parsnip is a native of Arabia; the sunflower was brought from Peru; spinach was first cultivated in Arabia; the horse-chestnut is a native of Thibet; the quince came from the island of Crete; the pear is supposed to be Egyptian origin; the horse-radish came from the South of Europe.

